# Socioeconomic inequalities in metabolic syndrome in the French West Indies

**DOI:** 10.1186/s12889-019-7970-z

**Published:** 2019-12-03

**Authors:** Zoé Colombet, Marlène Perignon, Benoît Salanave, Edwige Landais, Yves Martin-Prevel, Benjamin Allès, Sophie Drogue, Marie Josèphe Amiot, Caroline Méjean

**Affiliations:** 1 UMR 1110 MOISA, Univ Montpellier, CIRAD, CIHEAM-IAMM, INRA, Montpellier SupAgro, 2 place Pierre Viala, F-34000 Montpellier, France; 2 Nutritional Surveillance and Epidemiology Team (ESEN), French Public Health Agency, Paris-13 University, Centre de recherche en épidémiologie et statistiques, COMUE Sorbonne Paris Cité, Bobigny, France; 3 UMR204-Nutripass, French National Research Institute for Sustainable Development (IRD), Université de Montpellier, Montpellier, France; 4 Université Paris 13, Sorbonne Paris Cité, Equipe de Recherche en Epidémiologie Nutritionnelle (EREN), Centre d’Epidémiologie et Statistiques Paris Nord, Inserm (U1153), Inra (U1125), Cnam, Université Paris 5, Université Paris 7, F-93017 Bobigny, France

**Keywords:** Metabolic syndrome, Socioeconomic inequalities, Diet quality, French West Indies, Caribbean, Food consumption

## Abstract

**Background:**

Obesity and metabolic diseases represent a major health burden in the Caribbean, particularly since a large part of the population is disadvantaged. However, socioeconomic inequalities in chronic diseases are poorly explored in this region. We investigated the association between socioeconomic position and metabolic syndrome (MetS) prevalence and explored the contribution of diet quality to explain this association, among adults in the French West Indies.

**Methods:**

This cross-sectional analysis included 1144 subjects (≥16 y) from a multistage sampling survey conducted in 2013–2014 on a representative sample of the Guadeloupean and Martinican population. MetS prevalence was assessed using the Joint Interim Statement. Dietary intakes were estimated from 24 h-dietary recalls, and diet quality was assessed through the Diet Quality Index-International (DQI-I). Associations between socioeconomic indicators (education, employment, social assistance benefits) and MetS prevalence, and the potential contribution of diet quality in this association were assessed using multivariable logistic regression models, adjusted for sociodemographic characteristics.

**Results:**

MetS prevalence adjusted for age and sex was 21 and 30% among Guadeloupean and Martinican, respectively. Compared to high-educated participants, low-educated subjects were more likely to be at risk of MetS (OR = 2.4; 95%CI = [1.3–4.4], respectively), as were recipients of social assistance benefits compared to non-recipients (OR = 2.0; 95%CI = [1.0–4.0]). The DQI-I explained 10.5% of the overall variation in MetS due to education.

**Conclusions:**

Socioeconomic inequalities in MetS prevalence, reflected by education and social assistance benefits, were found. However, diet quality contributed only to socioeconomic inequalities due to education underlining that education may impact health through the ability to generate overall dietary behavior, long-term beneficial. Our work identified subgroups with higher risk of MetS, which is needed when implementing public health measures, particularly in this Caribbean population with of high poverty rates. Further prospective studies are needed to improve our understanding of the mechanisms of social inequalities in MetS in a high poverty rates context.

## Background

Studies in the Caribbean, including the French West Indies, though still few in number, highlight some urgent public health issues: a shift towards unhealthy dietary patterns and increasing rates of obesity and chronic diseases have been observed over recent decades, revealing an advanced phase in the nutrition transition [1–5]. Metabolic syndrome (MetS) is a high-risk condition associated with diabetes and with overall and cardiovascular mortality, thus reducing MetS prevalence could lessen the burden of those chronic diseases [6, 7]. MetS is a cluster of metabolic abnormalities: abdominal obesity, high blood levels of triglycerides, low high-density lipoprotein (HDL) cholesterolemia, high blood pressure and high fasting glucose [6]. Using the American Heart Association/National Heart, Lung, and Blood Institute (AHA/NHLBI) definition [7], prevalence of MetS in Caribbean adults is quite high: 18% in Jamaica [8], 29% in the Grenadian islands of Carriacou and Petite Martinique [3] and 43% in the San Juan Metropolitan Area of Puerto Rico [9]. In the French West Indies, MetS prevalence in the general population is unknown, but high prevalence of the different MetS traits was observed: 23 and 22% for obesity, 29 and 28% for hypertension, and 9 and 8% for pharmacologically-treated diabetes in Guadeloupe and Martinique, respectively [10–12].

In previous epidemiological studies, an inverse association between MetS and socioeconomic indicators such as education and income was often observed, particularly in women [13–18]. Evidence regarding employment status is more mixed [14, 15]. Very few studies assessing socioeconomic difference in MetS have been conducted in Caribbean populations. Studies in the San Juan Metropolitan Area of Puerto Rico showed an inverse association of MetS prevalence with education, and no association with marital status in women [19, 20]. By contrast, positive associations between prevalence of MetS and higher education and income were found in Jamaican men but not in women [8]. This finding may reflect the early stage of Jamaica’s epidemiological transition in the 90s and the situation may have changed since then. None of these studies examined simultaneously the three most frequently used socioeconomic indicators: education, employment status and income. Yet, they are generally weakly correlated suggesting some shared association but also that they are conceptually distinct, and their influence is transmitted by different social processes [21–23]. In fact, education is linked to health through knowledge, attitudes and skills while income reflects financial means and occupation can represent one’s social network [21]. Thus, these socioeconomic markers are not interchangeable and can have additive or synergistic effects on health [21, 23]. They should therefore be taken into account simultaneously to highlight the distinct socioeconomic facets that influence MetS [23]. Hence the identification of subgroups in Caribbean populations who are at higher risk of MetS is a key element when implementing public health measures, particularly in the current Caribbean context of widespread poverty and social inequalities in chronic diseases [24].

Concerning dietary factors, an inverse association between diet quality and MetS prevalence and incidence has been reported [25–29]. In addition, high socioeconomic position is consistently associated with high diet quality, underlining the importance of understanding the contribution of diet in socioeconomic inequalities in MetS. The aim of our study was to assess the association between socioeconomic indicators and MetS prevalence in adults (≥16 years) in the French West Indies, and to investigate the contribution of diet quality on the relationship between socioeconomic position and MetS.

## Methods

### Population

The subjects were participants aged 16 and over in the cross-sectional “Kannari survey: Health, Nutrition and Exposure to Chlordecone in the French West Indies”, conducted on Guadeloupean and Martinican adults and children by Santé Publique France (the French public health agency) in 2013–2014 [30, 31]. The Kannari study, aimed to be representative, was based on a multistage stratified random sample of the populations living in two locations, Guadeloupe and Martinique, to describe chlordecone food exposure and impregnation, health status and food intakes in these populations. Sample selection was based on a three-stage cluster design (geographic areas, household and individuals in the household), stratified by chlordecone contamination areas (coastline and inland).

The Kannari survey was conducted according to the Declaration of Helsinki guidelines, and the survey protocol received approval from the ethical research committee for South-West and Overseas II (Comité de protection des personnes Sud-Ouest et Outre-mer II, CPP No. 2–13-10) and the French Data Protection Authority (Commission Nationale Informatique et Libertés No. 913236). Informed consent was obtained from all the subjects.

### Data collection

Demographic and socioeconomic characteristics, health status and food frequency data were collected through face-to-face interviews at home using standardized questionnaires. Anthropometric data and blood pressure were also measured at participants’ homes. Dietary data were collected by phone by trained dietitians. For adults aged 18 and over who had given their consent, a biological sample was collected either at home by a nurse, or in a laboratory, at the subject’s choice.

#### Assessment of demographic and socioeconomic characteristics

Socioeconomic markers were education, employment status, and being a recipient or not of social assistance benefits and demographic characteristics were sex, age, location (Guadeloupe or Martinique), single-parent household, presence or not of at least one child in the household. As income information was not available, to be recipients of social assistance benefits was used to identify the most deprived participants in our sample [32], in the form of a guaranteed minimum income. Age ranges were 16–45 years, 46–60 years and over 60 years. Level of education was broken down into three categories according to the highest qualification attained: low (no or primary school), middle (below high school) and high (equivalent to or higher than high school). Employment status had three categories: unemployed and never-employed (unemployed, disabled, homemakers and students), employed and retired.

#### Dietary assessment

Dietary data were collected using two non-consecutive randomly assigned 24 h dietary recalls. Participants were asked to describe in detail their food intake (including composition of homemade recipes) and quantities consumed during the 24 h preceding the interview. Portion sizes were estimated using standard measurements (e.g. home containers, grams indicated on the package) or a validated illustrated booklet [33], representing more than 250 foods specific to the French West Indies (corresponding to 1000 generic foods) served in seven different portion sizes. In addition to 24 h recalls, participants completed a food frequency questionnaire (FFQ), about their usual frequency of consumption of 119 food and beverage groups over 12 months. As one aim of the Kannari study was to describe chlordecone food exposure and impregnation, the FFQ specifically included food groups contributing to chlordecone exposure such as seafood. More details about methods of food intake data collection used in the Kannari survey are published elsewhere [30]. Values for energy, macronutrients and micronutrients were estimated using published nutrient databases [34] and were extended for French West Indian market foods and recipes. The Multiple Source Method (MSM) was used to estimate usual dietary intake [35]. With the MSM, usual dietary intakes were estimated using the amounts of consumption from 24 h dietary recalls combined with consumption frequencies declared in the FFQ, taking into account inter- and intra-individual variations, according to sex and age.

Energy-underreporting participants were identified by the method proposed by Black [36] and excluded from the analyses. Briefly, basal metabolic rate (BMR) was estimated using Mifflin equations [37], since a high prevalence of overweight and obesity was observed in our study sample. BMR was compared to energy intake, taking into account a physical activity level of 1.55 to identify underreporters [36]. Subjects who reported specific conditions that could objectively explain low energy intake, such as a low-energy diet to lose weight or acute disease, were not recorded as underreporters.

#### Diet quality

To evaluate the overall quality of the diet, we used the Diet Quality Index-International (DQI-I) developed by Kim et al.*,* as it assesses several aspects of diet quality and allows international comparisons [38]. The DQI-I (range 0–100 points, 0 being the poorest and 100 being the highest possible score), including both nutrient- and food-group items, consists of 17 components grouped into four main categories: variety (overall food group variety; within-group variety for protein source), adequacy (vegetables, fruits, cereals, fiber, protein, iron, calcium, vitamin C), moderation (total fat, saturated fat, cholesterol, sodium, empty-energy foods) and overall balance (macronutrient ratio; fatty acid ratio). The cut-offs used for adequacy and moderation were those corresponding to United States Dietary Reference Intakes (DRIs) [39–41].

#### Health status

Participants were asked about their health status including whether a physician had diagnosed hypercholesterolemia, diabetes or hypertension, and their use of medication for these diseases. To reflect subject’s health status awareness, a binary variable was created: “has been diagnosed for at least one of these diseases or not”.

Weight was measured to the nearest kilogram on an electronic digital scale (SECA®), tared once a week, with the participant lightly dressed and shoeless. Height was measured using a portable gage (SOEHNLE®). Waist circumference was measured with a tape measure. Blood pressure was measured in subjects after a 5 min rest using an automatic validated device (Omron 750 CP2): the mean of two consecutive measurements at a 1 min interval was recorded. Body mass index (BMI) was calculated and categorized according to the World Health Organization (WHO) classification [42] and recoded into three categories: underweight or normal weight, overweight and obese.

For participants who agreed to blood sampling, fasting glucose, triglycerides and HDL-cholesterol concentrations were measured.

Physical activity level was assessed only in Martinicans using the validated Recent Physical Activity Questionnaire (RPAQ) which evaluate physical activity during the past 4 weeks across four domains: leisure time, occupation, commuting, and domestic life [43]. Estimates of the physical activity energy expenditure (PAEE) for the four domains were assessed by multiplying participation (h/d) by the metabolic cost of each activity, expressed in Metabolic Equivalent Task (MET) [44]. Weekly energy expenditure was estimated by computing each estimates of PAEE and then three levels of physical activity were defined: low, moderate, and high.

#### Definition of MetS

Prevalence of MetS was determined according to the Joint Interim Statement [6], the latest harmonizing definition, as meeting at least three of the following five criteria: 1) elevated waist circumference (≥94 cm for men and ≥ 80 cm for women), 2) elevated triglycerides (≥150 mg/dL or drug treatment for elevated triglycerides), 3) low HDL-cholesterolemia (< 40 mg/dL for men and < 50 mg/dL for women or dyslipidemia treatment), 4) elevated blood pressure (systolic blood pressure ≥ 130 mmHg and/or diastolic ≥85 mmHg or antihypertensive drug treatment) and 5) elevated fasting glucose (≥100 mg/dL or antidiabetic medication). Waist circumference and blood pressure were measured for all participants, but biological data were available only for a subsample. All the subjects were asked about medication for dyslipidemia, hypertension and diabetes.

### Statistical analysis

Comparisons between included participants and energy-underreporting subjects, according to location (Guadeloupe or Martinique) and according to MetS status were performed using Pearson’s or Rao-Scott’s chi-square tests, Fisher’s exact test or Student’s *t* test as appropriate.

Independent associations between each socioeconomic indicators and MetS prevalence were examined using multivariable logistic regression models. For each socioeconomic indicator (education, to be recipient of social benefits and employment), models adjusted for demographic factors such as location (Martinique or Guadeloupe), age, sex, single-parent household, presence of at least one child in the household and BMI were performed. Then, the three socioeconomic indicators and adjusted factors were included together in a logistic regression model, named base model, assessing the independent associations of socioeconomic indicators with the risk of MetS. Collinearity between socioeconomic indicators was investigated by examining the variance inflation factor, with a value of 4 as the maximum level to identify collinearity [45]. We then verified that DQI-I was significantly associated with MetS and with socioeconomic indicators using logistic and linear regression models, as appropriate. Secondly, a logistic regression model assessing the contribution of dietary quality on the association between socioeconomic indicators and the risk of MetS was performed adjusted for location (Martinique or Guadeloupe), age, sex, single-parent household, presence of at least one child in the household and BMI. Causal inferences regarding a possible mediating effect of diet quality must be viewed with caution, due to the cross-sectional design of our study. Individuals with hypertension, diabetes or hypercholesterolemia may change their diet after learning of their disease [46], by reducing their intake of unhealthy foods and may have a higher diet quality score compared with those with normal blood pressure, glycaemia or cholesterolemia values. Thirdly, a fully adjusted logistic regression model was therefore performed by adding variable related to health status awareness (self-reported diabetes, hypercholesterolemia and hypertension).

To assess the contribution of diet quality on socioeconomic differences in MetS, we measured the potential mediating effect of the diet quality through two indicators. First, the magnitude of change due to diet quality, assessed by the percentage change in the odds ratios (ORs) of the socioeconomic indicators computed as [(OR base model − OR model with mediator) / (OR base model − 1)] × 100 [47, 48]. Then, we went on to calculate the percentage reduction in deviance attributable to socioeconomic indicators accounted for by inclusion of the DQI-I. This reduction in deviance related to socioeconomic indicators, used as an overall statistical test of the mediating effect, quantifies the percentage of socioeconomic disparity for the risk of MetS explained by the DQI-I, named below mediator [48]. The deviance of the model is the mathematical function that compares the observed values of the response variable to those predicted by the model. The percentage reduction of deviance (RD) due to socioeconomic indicators explained by inclusion of the mediating factor was calculated as [(RD due to socioeconomic indicators in base model) − (RD due to socioeconomic indicators in model with mediator) / RD due to socioeconomic indicators in base model] × 100 [48].

To optimize the robustness of the statistical tests, we performed sensitivity analyses. First, to explore whether socioeconomic differences in MetS varied with age, interaction between age and socioeconomic indicators was tested, and the analyses were stratified according to median age: 53 years or less and over 53. Also, associations of physical activity level with DQI-I and MetS were assessed in Martinicans (data on physical activity were available only in Martinique), and the mediating effect of physical activity level on the association between socioeconomic position and MetS was investigated. Finally, we examined whether representing overall diet by nutrient and food intakes instead of DQI-I would better mediate the association between socioeconomic indicators and MetS. For sensitivity analyses, we used an approach identical to that described above.

To take into account the complex survey design in all the analyses, weighting was calculated. As one of the objective of the Kannari survey was to assess exposure to chlordecone and the sample selection was stratified by chlordecone contamination areas (coastline and inland), we take into account chlordecone contamination area in the weight calculation. Thus, weighting was calculated for each sex on age, education, marital status, birthplace, presence of at least one child in the household, living in an area with chlordecone contamination (coastline and inland) and urban size, using the iterative proportional fitting procedure according to the French national census reports [49]. We used specific survey procedures to take into account weighting and stratification.

For all analyses, a *p*-value of < 0.05 was considered statistically significant. Data management and statistical analyses were performed using SAS (version 9.3; SAS Institute, Inc., Cary, NC, USA.).

## Results

**Fig. 1:**
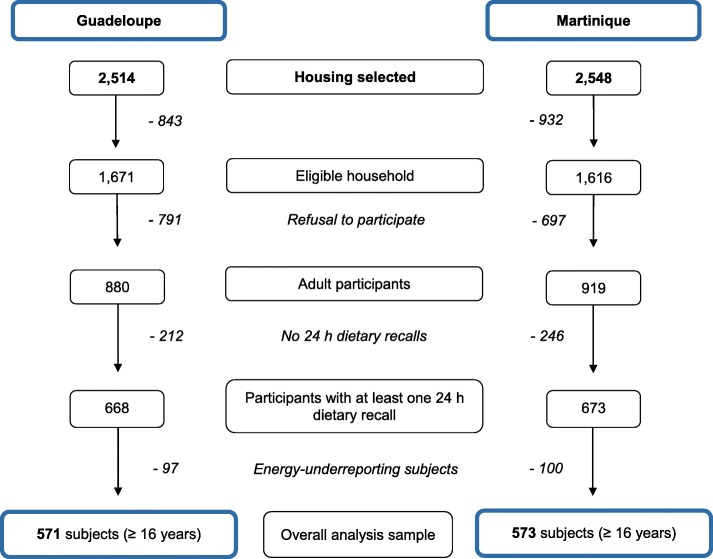
Flow-chart for inclusion of subjects in the final sample of the study (*n* = 1144)

Among the 1799 subjects who participated in the Kannari study, 1341 had at least one 24 h dietary recall (Fig. 1). We excluded 197 energy-underreporting subjects, leaving 1144 subjects (≥16 y) in the analysis sample.

Included subjects were older than energy-underreporting excluded subjects (mean 52.9 years (SD: 17.0) vs. 48.5 years (SD: 15.4); *p* < 0.001), and their prevalence of obesity was lower (Additional file 1: Table S1).

The prevalence of MetS was 23% in the analysis sample, and the prevalence of obesity was 21% (Table 1). More than half of the sample were women, and approximately 44% of subjects were aged 16–45 years. Regarding socioeconomic characteristics, 32% of the participants were unemployed or never-employed, 19% received social assistance benefits and 44% were low-educated. Almost 40% of the subjects were living with at least one child in their household and 6% were part of a single-parent household. The mean DQI-I was 60.8 points (SEM: 0.4) and ranged from 38.8 to 81.9 points. No difference according to location was observed except for MetS prevalence, which was markedly higher in Martinique than in Guadeloupe, the percentage of single-parent households, which was twice as high in Guadeloupe as in Martinique, and the mean DQI-I, which was higher in Guadeloupe (Table 1). After adjusting for age and sex, the prevalence of MetS was 21% (SEM: 0.02) in Guadeloupe and 30% (SEM: 0.03) in Martinique. In the subsample with no missing biological data (*n* = 534), the prevalence of MetS was 36% (30% in Guadeloupe and 40% in Martinique).

**Table 1:** Characteristics of the sample of Guadeloupe and Martinique subjects (≥16 y) from the Kannari study (*n* = 1144)^a^

	Total (*n* = 1144)	Guadeloupe (*n* = 571)	Martinique (*n* = 573)	*P* ^b^
	%	%	%	
Sex				0.91
Men	43.0	42.7	43.2	
Women	57.0	57.3	56.8	
Age class				0.22
16–45 years	44.3	47.8	41.0	
46–60 years	28.6	25.6	31.5	
> 60 years	27.1	26.7	27.5	
Employment status				0.67
Unemployed, disabled, homemakers or students	32.3	33.4	31.2	
Active	43.3	43.9	42.8	
Retired	24.4	22.7	26.0	
Education				0.43
Low	44.4	43.4	45.4	
Middle	18.6	17.0	20.2	
High	37.0	39.6	34.4	
Receive social assistance benefits	18.7	19.1	18.3	0.84
At least one child in the household	38.6	42.0	35.2	0.14
Single-parent household	6.1	8.1	4.3	**0.04**
Metabolic syndrome	22.8	17.7	27.9	**0.003**
Elevated waist circumference (men ≥ 94 cm / women ≥ 80 cm)	58.9	57.5	60.3	0.54
Elevated triglycerides	8.2	4.2	12.1	**< 0.001**
Reduced HDL-cholesterol HDL	17.6	15.5	19.7	0.21
Elevated blood pressure (≥130/85 mmHg)	56.4	55.2	57.6	0.60
Elevated fasting glucose	20.7	18.4	23.1	0.17
Body mass index class				0.07
Underweight or normal weight	45.1	49.1	41.0	
Overweight	33.5	33.4	33.7	
Obese	21.4	17.5	25.2	
	Mean ± SEM	Mean ± SEM	Mean ± SEM	
Diet Quality Index - International (DQI-I) (0–100 points)	60.8 ± 0.4	62.0 ± 0.5	59.6 ± 0.5	**0.001**
Moderation (0–30 points)	17.1 ± 0.2	17.4 ± 0.2	16.9 ± 0.2	0.18
Variety (0–20 points)	16.5 ± 0.1	16.9 ± 0.2	16.0 ± 0.2	**< 0.001**
Adequacy (0–40 points)	26.1 ± 0.2	26.6 ± 0.3	25.7 ± 0.3	**0.03**
Overall balance (0–10 points)	0.3 ± 0.1	0.4 ± 0.1	0.3 ± 0.1	0.35

Values are presented as percentage or mean ± standard error of the mean (SEM)

^a^ Sex-specific data weighted for education, marital status, birthplace, presence of at least one child in the household, living in an area with chlordecone contamination (coastline and inland) and urban size, using the 2012 national census

^b^ Student’s *t* test or chi-square test as appropriate (significant values are bold)

In univariable models, the DQI-I was lower in unemployed or never-employed subjects (β = − 2.9; 95%CI = [− 4.5; − 1.3]) and recipients of social assistance benefits (β = − 3.4; 95%CI = [− 5.0; − 1.7]) than in employed subjects and non-recipients, and higher in retired (β = 5.9; 95%CI = [4.3; 7.4]) and low-educated participants (β = 3.1; 95%CI = [1.5; 4.7]) than in employed and high-educated subjects (Additional file 1: Table S2). Also, the DQI-I was positively associated with MetS (β = 3.7; 95%CI = [2.1; 5.4]).

Associations between socioeconomic indicators and MetS, are reported in Additional file 1: Table S3. Adjusted for demographic characteristics and BMI, low-educated participants were more likely to be at risk of MetS than high-educated participants, as recipients of social assistance benefits compared with non-recipients. Also, unemployed or never-employed individuals were more likely to be at risk of MetS compared with employed subjects. However, adjusted for other socioeconomic indicators, employment status became non-significant (Table 2). Risk of MetS according to categories of social assistance benefits and education levels were attenuated when adjusted for the other socioeconomic indicators, compared with previous models (Table 2).

**Table 2:** Associations between metabolic syndrome and socioeconomic indicators in Guadeloupe and Martinique subjects (≥16 y) from the Kannari study (*n* = 1144)^a^

	Base model ^b^			Model assessing the mediating effect ^c^			%VOR^e^	%RD^f^	Fully adjusted model ^d^			%VOR^e^	%RD^f^
	OR	CI95%	*p*	OR	CI95%	*p*			OR	CI95%	*p*		
Employment status			0.59			0.43					0.29		
Unemployed, disabled, homemakers or students	1.39	[0.74; 2.61]		1.51	[0.80; 2.84]				1.76	[0.85; 3.64]			
Active		Reference			Reference					Reference			
Retired	1.06	[0.42; 2.65]		1.02	[0.42; 2.51]				1.02	[0.44; 2.19]			
Education			**0.02**			**0.02**					0.05		
Low	**2.40**	[1.31; 4.36]		**2.30**	[1.27; 4.17]		7.14	10.45	**2.11**	[1.15; 3.86]		20.71	33.10
Middle	1.65	[0.81; 3.35]		1.60	[0.78; 3.25]				1.37	[0.64; 2.93]			
High		Reference			Reference					Reference			
Recipients of social assistance benefits	**2.04**	[1.04; 4.00]	**0.04**	**2.07**	[1.06; 4.05]	**0.03**	−2.88		1.76	[0.79; 3.93]	0.17	26.92	45.45
Diet Quality Index – International (DQI-I) overall				1.04	[1.004; 1.07]	**0.03**			1.02	[0.99; 1.06]	0.17		

OR: odds ratio; 95% CI: 95% confidence interval

^a^ Sex-specific data weighted for education, marital status, birthplace, presence of at least one child in the household, living in an area with chlordecone contamination (coastline and inland) and urban size, using the 2012 national census

^b^ Base model: multivariable model with the three socioeconomic factors, adjusted for location (Martinique or Guadeloupe), age, sex, single-parent household, presence of at least one child in the household and body mass index

^c^ Model assessing the mediating effect: base model + overall DQI-I

^d^ Fully adjusted model: base model + overall DQI-I, adjusted for health diagnosis

^e^ %VOR: percentage variation in OR; ((OR_basemodel_ − OR_model with mediator_) / (OR_basemodel_ − 1)) × 100

^f^ %RD: percentage reduction of deviance due to socioeconomic indicators explained by inclusion of mediator ((reduction in deviance due to socioeconomic indicators of base model) − (reduction in deviance due to socioeconomic indicators of base model + mediator) / reduction in deviance due to socioeconomic indicators of base model) × 100

Adding the DQI-I to the model reduced the OR for MetS by 7% in low-educated subjects and it explained about 10.5% of the overall variation due to education in MetS i.e. reduction in deviance due to education (Table 2). Adding the DQI-I increased the OR of the association between social benefits assistance and MetS and therefore it cannot be considered as a mediating factor of this association. When health diagnosis was added, diet quality and health diagnosis together explained about 33% of the overall variation due to education in MetS and 45% of the variation due to social assistance benefits. Representing diet as intakes of food groups in sensitivity analyses have also shown a mediating effect on the relationship with social assistance benefits: intakes of food groups explained 20% of the overall variation due to social assistance benefits and 12% of the overall variation due to education. Representing diet as intakes of nutrient did not explain as much of the variation due to education as the DQI-I (5% of the overall variation due to education), but explained variation due to social assistance benefits: 15% of the variation due to social assistance benefits.

Sensitivity analyses, conducted in Martinican subjects, showed that physical activity level slightly mediated the association between socioeconomic indicators and MetS: 16% for education (OR_low vs. high_ = 2.3; 95%CI = [1.0; 5.0] to OR_low vs. high_ = 2.1; 95%CI = [1.0; 4.7] and OR_middle vs. high_ = 1.8; 95%CI = [0.7; 4.3] to OR_middle vs. high_ = 1.8; 95%CI = [0.7; 4.2]) and 9% for recipients of social assistance benefits (OR = 2.8; 95%CI = [1.2; 6.2] to OR = 2.7; 95%CI = [1.2; 5.9]). Finally, in analyses stratified according to median age (≤ 53 y and > 53 y), associations with education and being recipients of social assistance benefits remained significant only among younger participants. In participants aged 53 and under, the DQI-I explained 13% of the overall variation due to education in MetS and 5% of the overall variation due to social assistance benefits.

## Discussion

The present study conducted in representative populations in Guadeloupe and Martinique, highlights socioeconomic inequalities in MetS in an unfavorable socioeconomic context. In this sample of Guadeloupeans and Martinicans, diet quality slightly contributed to the differences in the risk of MetS according to education.

The MetS prevalence observed in our sample was lower in Guadeloupe than in Martinique (18% vs. 28%), due to lower prevalence of abdominal obesity in Guadeloupean men than in Martinican men (respectively 30% vs. 46%, *p* = 0.02). MetS prevalence was lower than that observed in previously published Caribbean studies conducted in the Grenadian islands of Carriacou and Petite Martinique (29%) [3] and in the San Juan Metropolitan Area of Puerto Rico (43%) [9]. Yet these last two studies used the AHA/NHLBI definition of MetS, for which cut-offs for abdominal obesity are higher (waist circumference ≥ 102 cm for men and ≥ 88 cm for women) [7] than those used by the Joint Interim Statement. For comparison, in our study, MetS prevalence was 14% in Guadeloupe and 23% in Martinique with this definition.

Weak correlation was found between education, to be recipient of social benefits and employment suggesting that they are conceptually distinct, and their influence is transmitted by different social processes [21–23]. Our study showed that risks of MetS according to social assistance benefits and education in models adjusted for the two other socioeconomic position indicators remained significant, even they were slightly lower than in models not adjusted for other socioeconomic markers. Education and social assistance benefits therefore appear to be independent predictors of MetS. Low-educated subjects and recipients of social assistance benefits are more likely to be at risk of MetS than highly educated persons and non-recipients. Our findings are in line with the literature reporting an inverse association between socioeconomic status and MetS [13–18], and the Caribbean study conducted in Puerto Rico, which showed lower MetS prevalence in individuals with higher education, compared to those with lower education [20]. Health literacy, i.e. the degree to which individuals have the capacity to obtain, process, and understand basic health information and services needed to make appropriate health decisions [50] is shown to be higher in highly educated participants [51, 52]. This partly explains the inverse relationship between MetS and education level. Better educated participants may be more receptive to health and dietary messages and better able to access appropriate health services and communicate with them [21]. Also, in health studies, it has been shown that education captures the transition from parents’ socioeconomic position (received) to adulthood socioeconomic position (own) [53, 54]. It reflects material, intellectual, and other resources of the family of origin, begins at early ages, is influenced by access to and performance in primary and secondary school and reaches final attainment in young adulthood for most people. Therefore it captures the long term influences of early life circumstances on adult health [54, 55]. Receiving social assistance benefits may have a direct effect on health through limited material resources and financial means [21, 32]. Indeed, the higher risk of MetS among recipients of social assistance benefits may be explained by reduced access to healthy lifestyles such as physical leisure activities due to low financial resources that may cause them to forego health care. In contrast, employment status was no independently associated with MetS prevalence. After adjustment for other socioeconomic factors (education and receiving social assistance benefits), unemployed and never-employed subjects were no longer more likely to be at risk of MetS compared to employed participants. We could hypothesize that the difference observed in the risk of MetS according to the employment status was due to financial means, took into account when adjusted for social assistance benefits. Also, education level determines adult employment [54, 55], that may explained the relationships between employment status and MetS. The differences between unadjusted and adjusted for all the socioeconomic position indicators also suggest that the use of a single socioeconomic position measure leads to misinterpreting relations between socioeconomic position indicator and MetS and confirm that they should be studied simultaneously [21–23]. Under or overestimation of socioeconomic disparities in MetS may have implications for public health strategies. Analysis stratified according to median age showed that associations with education and being recipients of social assistance benefits remained significant only among younger participants (≤ 53 y), suggesting that low-socioeconomic groups are most likely to develop early-onset MetS. Our findings provide useful information to identify subgroups of the population at high risk. This is a key element to help the implementation of public health measures that target the disadvantaged groups, particularly in the current context of health inequalities that still remain important.

In the French West Indies, socioeconomic differences in MetS that might lead to social inequalities in cardiovascular morbidity and mortality seemed to be driven by education and to be recipient of social benefits. It is therefore necessary to identify modifiable factors, such as dietary intake, involved in socioeconomic differences in MetS to gain a better understanding of the mechanisms driving social inequalities in health. However, our study showed that diet quality explained only 10.5% of the overall variation due to education in MetS and did not explained variation due to social assistance benefits. Analysis stratified according to median age showed that diet quality explained the overall variation due to education in MetS and slightly explained the variation due to social assistance benefits in younger subjects (≤ 53 y) but not in older ones, underlining the contribution of the diet quality in socioeconomic differences in early-onset MetS. Intakes of nutrient and food groups also slightly contributed to the educational disparities in MetS whereas they contributed to the variation due to social assistance benefits, unlike the DQI-I. Hence limited financial resources, as assessed by receiving social assistance benefits, may influence the diet by reducing intakes of specific costly foods (fruits and vegetables, seafood) rather than overall diet, whereas education may have an effect on all the dimensions of dietary quality, as it is associated with ability to generate overall dietary behavior that is beneficial in the long term. Also, physical activity level in Martinicans explained 9% of the overall variation due to social assistance benefits in MetS, that may be explained by a reduced access to physical leisure activities due to low financial resources.

The weak mediating effect of diet quality in socioeconomic differences in MetS due to education may be explained by the positive association between diet quality and MetS. Individuals with MetS may adopt healthier dietary behaviors after being diagnosed for a disease [46]. Indeed, after adjustment for diagnoses of hypertension, diabetes and hypercholesterolemia, the association between DQI-I and MetS became non-significant, in line with the results of two previous studies [56, 57]. Diet quality contributed only to socioeconomic inequalities due to education underlining that education may impact health through health literacy, especially the ability to understand health and dietary information and adopt new dietary behavior after being diagnosed.

The interpretation of the present results should take into account several limitations. First, an inherent limitation of a cross-sectional design is the impossibility to infer causal relationships. In addition, the rather small size of our sample limit the statistical power and may question about the generalizability of our findings. However, the Kannari survey was carefully designed to be representative of Guadeloupean and Martinican and analyses were weighted according to French national census data, which allow bias to be limited. Another limitation of our work is that biological data were available only in a subsample of volunteer adults (≥18 years). In the subsample with no missing biological data, MetS prevalence was higher. However, individuals in this subsample were older on average than the subjects in the whole sample, suggesting an overestimation of MetS prevalence. Also, we used the latest harmonizing definition of MetS and applied recommended cut-offs for Caucasian and Sub-Saharan African for central obesity, which are lower than cut-offs in the AHA/NHLBI definition. This could have caused a slight overestimation of the prevalence of MetS in our sample. Moreover, 15% of the subjects were identified as energy-underreporters and excluded: compared with included subjects, the excluded individuals were younger and the percentage of unemployed or never-employed individuals was higher, which may have caused an underestimation of socioeconomic disparities in our analysis sample. Also, as information about income was not available, social assistance benefits was used as a marker of financial resources [32]. However, this marker only categorized participants according to very low resources or not instead of a gradient of resources that could have a gradual effect on the risk of MetS. Finally, we could not adjust for the impact of other types of mediators of socioeconomic inequalities in MetS like smoking status, cardiovascular history, adverse material and childhood circumstances and psychosocial factors [58], as they were not collected in the Kannari study. However, sensitivity analysis conducted in Martinicans showed that adjustment for physical activity did not modify our findings.

## Conclusions

In conclusion, socioeconomic inequalities in MetS, reflected by education and social assistance benefits, were found in two Caribbean populations. However, diet quality slightly contributed to socioeconomic inequalities due to education, while nutrient and food groups intake also contributed to socioeconomic inequalities due to social assistance benefits. Further prospective studies are now needed for a better understanding of the mechanisms of social inequalities in MetS in a context of high poverty rates in order to reduce health inequalities observed in the Caribbean.

## Supplementary information

**Additional file 1: Table S1.** Comparisons of individual characteristics between participants (*n* = 1144) and energy-underreporting subjects (*n* = 197) from the Kannari study subjects (≥16 y). **Table S2.** Associations between overall Diet Quality Index – International (DQI-I) and demographic and socioeconomic characteristics in Guadeloupe and Martinique subjects (≥16 y) from the Kannari study (*n* = 1144)*. **Table S3.** Associations between overall metabolic syndrome (MetS) and socioeconomic characteristics in Guadeloupe and Martinique subjects (≥16 y) from the Kannari study (*n* = 1144)*.

## Data Availability

The data that support the findings of this study have been provided by the French Public Health Agency (Santé publique France) and are not publicly available. Request have to be made to the French Public Health Agency.

## Abbreviations

95% CI: 95% confidence interval
AHA: American Heart Association
BMI: Body mass index
BMR: Basal metabolic rate
DQI-I: Diet Quality Index-International
ENNS: Etude Nationale Nutrition Santé
FFQ: Food frequency questionnaire
HDL: High-density lipoprotein
MET: Metabolic Equivalent Task
MetS: Metabolic syndrome
NHLBI: National Heart, Lung, and Blood Institute
OR: Odds ratio
PAEE: Physical activity energy expenditure
RD: Reduction of deviance
SD: Standard deviation
SEM: Standard error of the mean
