# Supplementary material for: Socioeconomic inequalities in metabolic syndrome in the French West Indies
Source: BMC Public Health. 2019 Dec 3;19:1620. doi: 10.1186/s12889-019-7970-z (PMC6888917; doi:10.1186/s12889-019-7970-z)
Supplement: Supplementary file 1 — Additional file 1: Table S1. Comparisons of individual characteristics between participants (n = 1144) and energy-underreporting subjects (n = 197) from the Kannari study subjects (≥16 y). Table S2. Associations between overall Diet Quality Index – International (DQI-I) and demographic and socioeconomic characteristics in Guadeloupe and Martinique subjects (≥16 y) from the Kannari study (n = 1144)*. Table S3. Associations between overall metabolic syndrome (MetS) and socioeconomic characteristics in Guadeloupe and Martinique subjects (≥16 y) from the Kannari study (n = 1144)*. [file 12889_2019_7970_MOESM1_ESM.docx]

**SUPPLEMENTAL MATERIAL**

**Socioeconomic inequalities in metabolic syndrome in the French West Indies**

**Running Title:** Caribbean social disparities in metabolic syndrome

Zoé Colombet^1^*, Marlène Perignon^1^, Benoît Salanave^2^, Edwige Landais^3^, Yves Martin-Prevel^3^, Benjamin Allès^4^, Sophie Drogue^1^, Marie Josèphe Amiot^1^, Caroline Méjean^1^

1. MOISA, Univ Montpellier, CIRAD, CIHEAM-IAMM, INRA, Montpellier SupAgro, Montpellier, France
2. Nutritional Surveillance and Epidemiology Team (ESEN), French Public Health Agency, Paris-13 University, Centre de recherche en épidémiologie et statistiques, COMUE Sorbonne Paris Cité, Bobigny, France.
3. UMR204-Nutripass, French National Research Institute for Sustainable Development (IRD), Université de Montpellier, Montpellier, France
4. Université Paris 13, Sorbonne Paris Cité, Equipe de Recherche en Epidémiologie Nutritionnelle (EREN), Centre d’Epidémiologie et Statistiques Paris Nord, Inserm (U1153), Inra (U1125), Cnam, Université Paris 5, Université Paris 7, F-93017, Bobigny, France

Colombet, Perignon, Salanave, Landais, Martin-Prevel, Allès, Drogue, Amiot, Méjean

**Corresponding author:** Zoé Colombet

INRA, UMR 1110 MOISA, 2 place Pierre Viala, F-34000 Montpellier, France

Phone number: 00 33 4 99 61 30 03 / Fax number: 00 33 4 99 61 25 51

E-mail: zoe.colombet@inra.fr

# **Table S1.** Comparisons of individual characteristics between participants (*n* = 1,144) and energy-underreporting subjects (*n* = 197) from the Kannari study subjects (≥16 y)

|  | **Participants (*n* = 1,144)** | **Underreporters (*n* = 197)** | ***p*** * |
| --- | --- | --- | --- |
|  | % | % |  |
| **Sex** |  |  | 0.98 |
| *Men* | 38.99 | 39.09 |  |
| *Women* | 61.01 | 60.91 |  |
|  |  |  |  |
| **Location** |  |  | 0.86 |
| *Guadeloupe* | 49.91 | 49.24 |  |
| *Martinique* | 50.09 | 50.76 |  |
|  |  |  |  |
| **Age class** |  |  | **0.01** |
| *16–45 years* | 31.82 | 41.62 |  |
| *46–60 years* | 33.74 | 34.52 |  |
| *>60 years* | 34.44 | 23.86 |  |
|  |  |  |  |
| **Employment status** |  |  | **0.03** |
| *Unemployed, disabled,  homemakers or students* | 27.19 | 34.01 |  |
| *Active* | 42.22 | 44.16 |  |
| *Retired* | 30.59 | 21.83 |  |
|  |  |  |  |
| **Education** |  |  | 0.38 |
| *Low* | 46.24 | 41.12 |  |
| *Middle* | 22.20 | 25.38 |  |
| *High* | 31.56 | 33.50 |  |
|  |  |  |  |
| **Receive social assistance benefits** | 13.99 | 17.26 | 0.23 |
|  |  |  |  |
| **At least one child in the household** | 29.90 | 41.62 | **0.001** |
|  |  |  |  |
| **Single-parent household** | 7.08 | 10.66 | 0.08 |
|  |  |  |  |
| **Body mass index class** |  |  | **<0.001** |
| *Underweight or normal weight* | 39.07 | 16.24 |  |
| *Overweight* | 36.45 | 37.56 |  |
| *Obese* | 24.48 | 46.19 |  |

Values are presented as percentages.

* Student’s *t* test or chi-square test as appropriate

# **Table S2.** Associations between overall Diet Quality Index – International (DQI-I) and demographic and socioeconomic characteristics in Guadeloupe and Martinique subjects (≥16 y) from the Kannari study (n = 1,144)*

|  | **Univariable** | | |
| --- | --- | --- | --- |
|  | **β** | **95% CI** | ***p*** |
| **Employment status** |  |  |  |
| *Unemployed, disabled, homemakers or students* | -2.86 | [-4.46 ; -1.27] | **<0.001** |
| *Active* | 1.00 |  |  |
| *Retired* | 5.86 | [4.32 ; 7.40] | **<0.001** |
|  |  |  |  |
| **Education** |  |  |  |
| *Low* | 3.09 | [1.48 ; 4.70] | **<0.001** |
| *Middle* | -0.46 | [-2.31 ; 1.38] | **0.62** |
| *High* | 1.00 |  |  |
|  |  |  |  |
| **Receive social assistance benefits** |  |  |  |
| *Yes* | -3.37 | [-5.03 ; -1.71] | **<0.001** |
| *No* | 1.00 |  |  |

95% CI: 95% confidence interval

* Sex-specific data weighted for education, marital status, birthplace, presence of at least one child in the household, living in an area with chlordecone contamination (coastline and inland) and urban size, using the 2012 national census.

# **Table S3.** Associations between overall metabolic syndrome (MetS) and socioeconomic characteristics in Guadeloupe and Martinique subjects (≥16 y) from the Kannari study (n = 1,144)*

|  | **Multivariable model** ** | | |
| --- | --- | --- | --- |
|  | **OR** | **CI95%** | ***p*** |
| **Employment status** |  |  | **0.04** |
| Unemployed, disabled, homemakers or students | **2.16** | [1.18 ; 3.97] |  |
| Active |  | Reference |  |
| Retired | 1.13 | [0.44 ; 2.94] |  |
| **Education** |  |  | **0.004** |
| Low | **2.78** | [1.52 ; 5.08] |  |
| Middle | 1.74 | [0.84 ; 3.60] |  |
| High |  | Reference |  |
| **Recipients of social assistance benefits** | **2.67** | [1.38 ; 5.17] | **0.004** |

OR: odds ratio; 95% CI: 95% confidence interval

* Sex-specific data weighted for education, marital status, birthplace, presence of at least one child in the household, living in an area with chlordecone contamination (coastline and inland) and urban size, using the 2012 national census.

** Multivariable model: adjusted for location (Martinique or Guadeloupe), age, sex, single-parent household, presence of at least one child in the household and body mass index
